# Design of a pyrolysis system and the characterisation data of biochar produced from coconut shells, carambola pruning, and mango pruning using a low-temperature slow pyrolysis process

**DOI:** 10.1016/j.dib.2023.109997

**Published:** 2023-12-21

**Authors:** Mohammad Shahid Shahrun, Mohammad Hariz Abdul Rahman, Nur Adliza Baharom, Fauzi Jumat, Mohamad Jani Saad, Mohd Fazly Mail, Norziana Zin Zawawi, Farah Huda Sjafni Suherman

**Affiliations:** a Soil & Fertilizer Research Centre, MARDI, Serdang, Selangor, 43400, Malaysia; b Agrobiodiversity & Environment Research Centre, MARDI, Serdang, Selangor, 43400, Malaysia; c Horticulture Research Centre, MARDI, Serdang, Selangor, 43400, Malaysia; d Engineering Research Centre, MARDI, Serdang, Selangor, 43400, Malaysia

**Keywords:** Agricultural wastes, Biochar, Pyrolysis, Self-heating process

## Abstract

Biochar production is an effective approach to managing abundant agricultural wastes. Pruning wastes from trimming the branches of trees such as carambola and mango, as well as coconut shells, are among the agricultural wastes that have reutilisation potential, which would simultaneously reduce the space required for disposal. In this study, the potential use of these wastes by converting them into biochar was investigated. The data presented in this study highlight the design of a pyrolysis system for a low-temperature slow pyrolysis process, as well as the characterisation data of the biochar produced using this system. The data collected included the elemental composition, porosity, as well as surface and adsorption characteristics of the biochar. These data indicate that the biochar produced had certain qualities that would enable its use for specific agricultural and industrial purposes. Meanwhile, the design indicated that it could facilitate small farms with specific outputs. In brief, these data can be used as references for developing a small-scale system for agricultural waste management using different types of crops.

Specifications Table

**Table:** 

Subject	Chemical engineering, carbon materials
Specific subject area	Pyrolysis, biochar production
Data format	Raw and analysed data
Type of data	Tables and figures
Data collection	Design and drawing of the pyrolyser: SolidWorks software version 2020. Elemental composition: elemental analyser (vario MACRO cube). pH and EC: Eutech P700 (Eutech Instruments). Ash content: standard ashing by incineration in a muffle furnace at 500 °C. Calorific content: samples outsourced to an accredited laboratory (MARDILab). Porosity test: Micromeritics® Tristar II Plus. Surface area analysis: Scanning Electron Micrography (SEM) using FEI Quanta 400. Adsorption study: jar test method with sample analysed using liquid chromatography–mass spectrometry (LC-MS/MS) (Sciex 5500).
Data source location	Malaysian Agricultural Research and Development Institute (MARDI), Serdang, Selangor, Malaysia (latitude: 2.99077383, longitude: 101.699415)
Data accessibility	Repository name: Mendeley Data Data identification number: 10.17632/d7hj9pdy32.2 Direct URL to data: https://data.mendeley.com/datasets/d7hj9pdy32/2
Related research article	Baharom, N. A., Rahman, M. H. A., Shahrun, M. S., Suherman, F. H. S., & Masdar, S. N. H. (2020). Chemical composition and antimicrobial activities of wood vinegars from carambola, coconut shells and mango against selected plant pathogenic microorganisms. Malaysian Journal of Microbiology, 16(6). https://doi.org/10.21161/mjm.190652

## 1 Value of The Data

• The data describe the characteristics of biochar produced from coconut shells, carambola pruning, and mango pruning using low-temperature slow pyrolysis with an upward pyrolysation process.
• Upward pyrolysation with self-heating is a common technique for producing biochar, especially among farmers in Malaysia. Therefore, the data could be compared with other available sources in the market.
• The data also provide valuable information about biochar qualities that would enable it to be used for specific agricultural uses. Users and farmers can benefit from the application of specific types of biochar.
• In addition to the widely used coconut shell biochar, biochar obtained from carambola and mango pruning demonstrated their potential use as added-value products due to the characteristics identified in this study.

## 2 Background

Biochar and wood vinegar production using the pyrolysis process has become popular due to the usefulness of the product for various applications, particularly in the agricultural sector. However, a design is needed that is applicable for smallholder farmers, and a study is required to characterise the data about the product made using this material. This study was conducted to develop a small-scale field pyrolyser that could be used to produce biochar from agricultural residues such as coconut shells, mango pruning, and carambola pruning. This was followed by a characterisation study to evaluate each of the products obtained using this process. The information can be used as references to guide stakeholders on the different qualities of the biochar produced using the system. The design and data could both be useful to farmers seeking to emulate the system for field biochar and wood vinegar production, as well as in selecting the raw materials best suited to eventual use in related agricultural activities.

## 3 Data Description

**Fig. 1:**
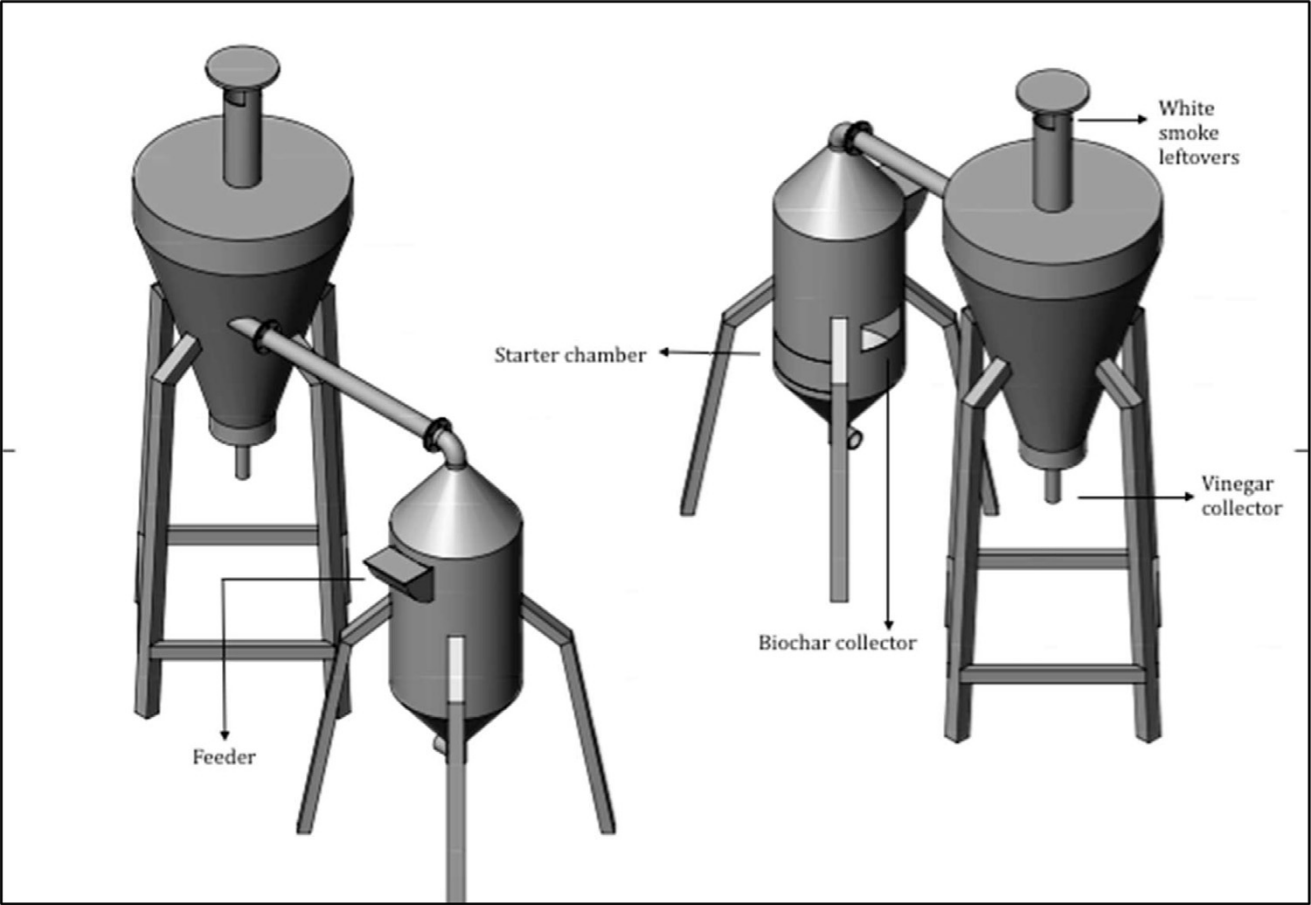
Overall design of the pyrolyser with two chambers for producing biochar and wood vinegar.

**Fig. 2:**
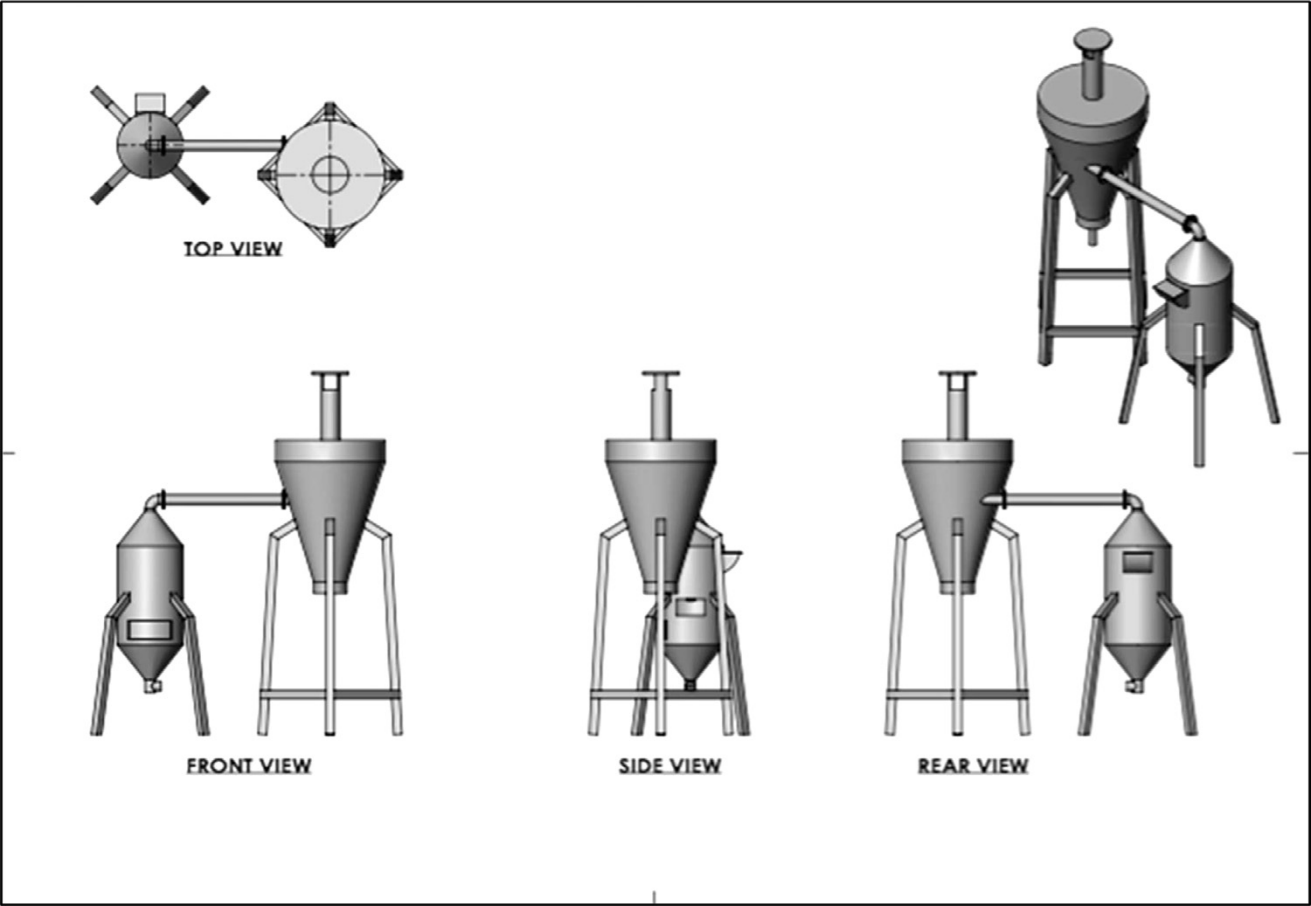
Different perspectives of the pyrolyser design: top, front, side, and rear views.

**Fig. 3:**
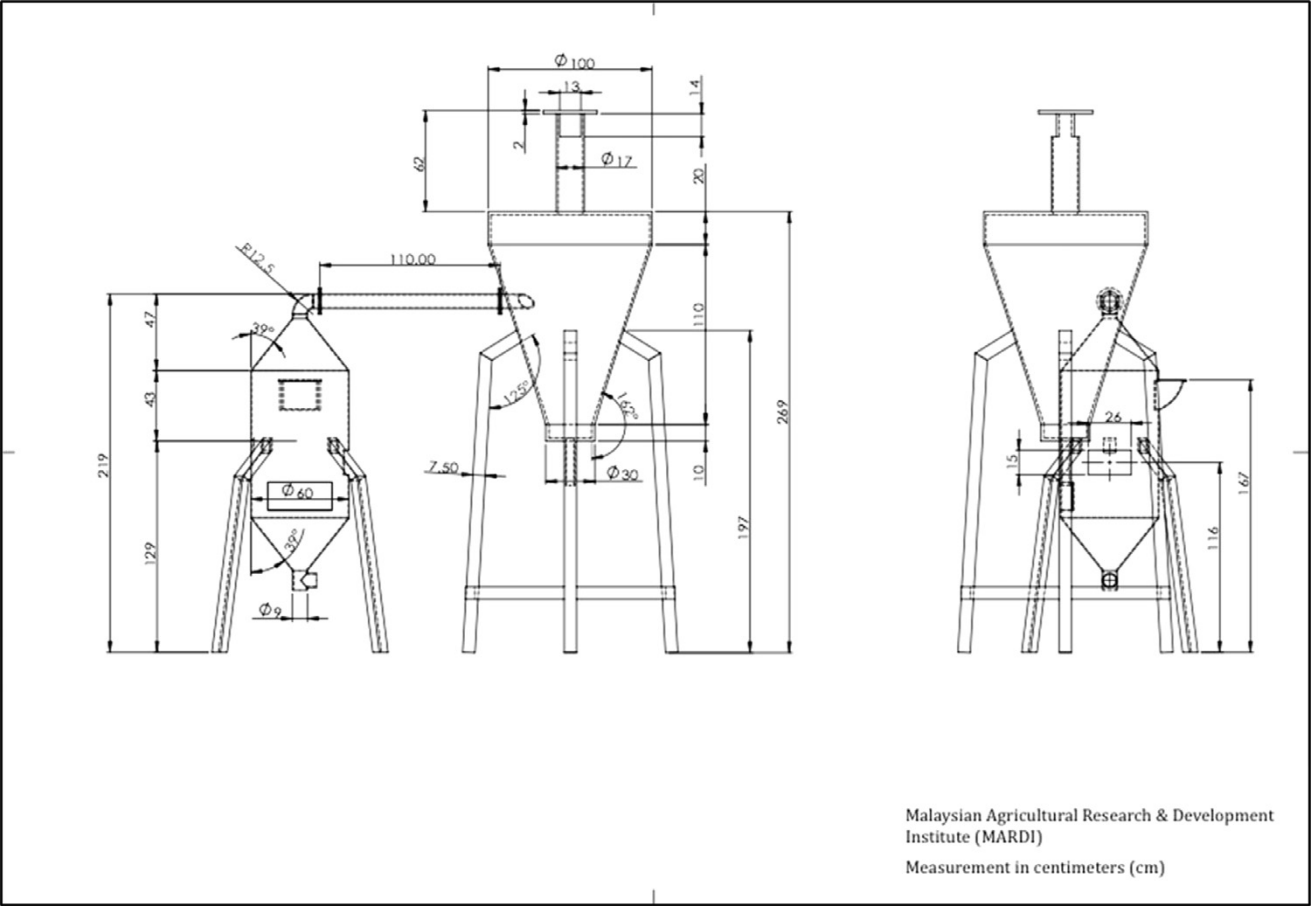
Schematic diagram of the pyrolyser design.

**Fig. 4:**
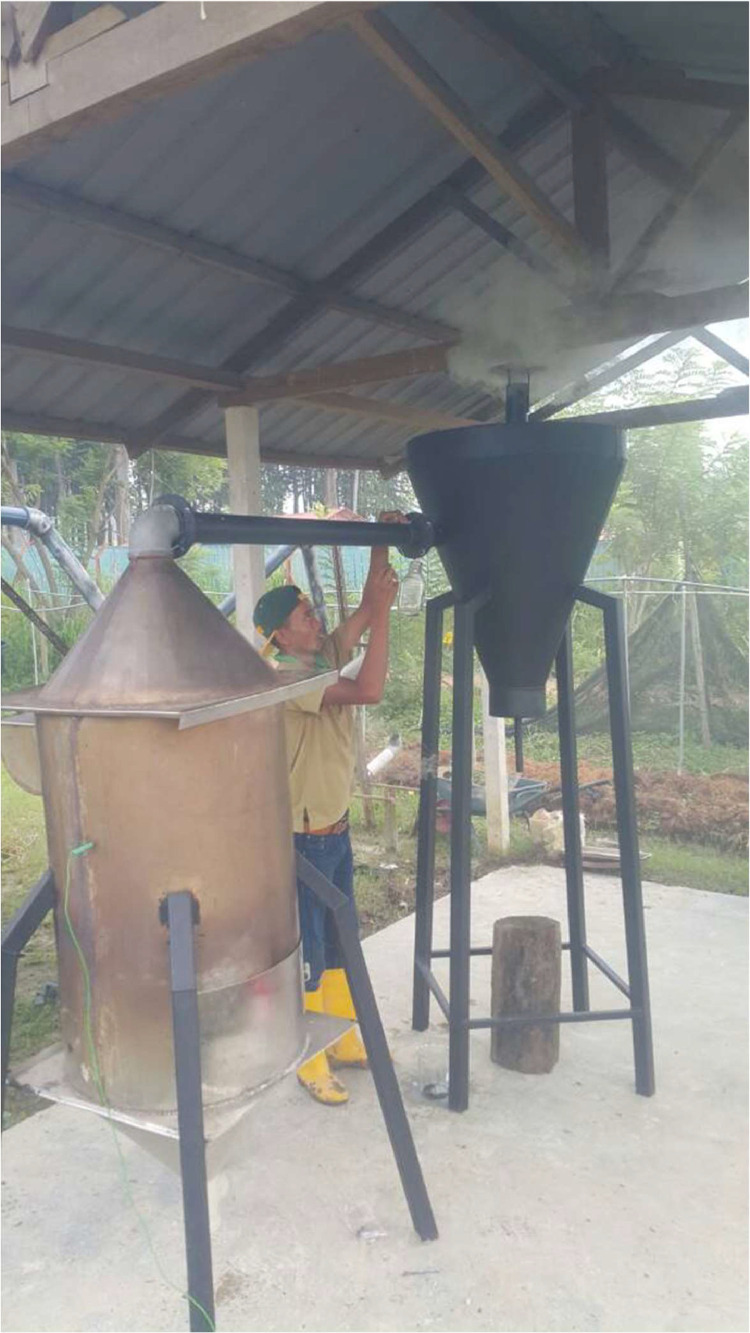
Actual view of the pyrolyser while in operation.

**Fig. 5:**
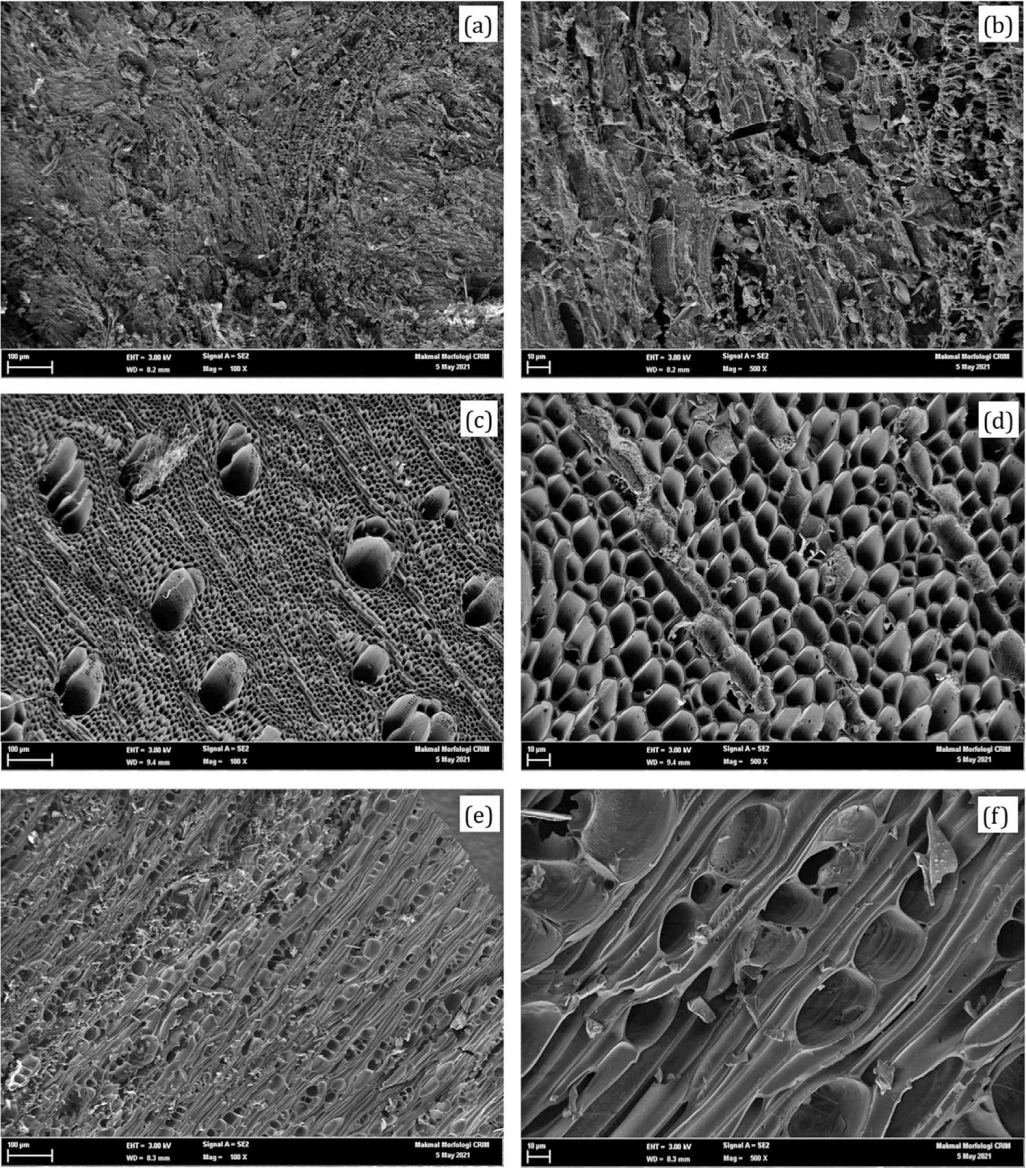
Scanning electron micrographs (SEM) of coconut shell biochar at magnifications of (a) 100X and (b) 500X; carambola pruning biochar at magnifications of (c) 100X and (d) 500X; and mango pruning biochar at magnifications of (e) 100X and (f) 500X.

**Fig. 6:**
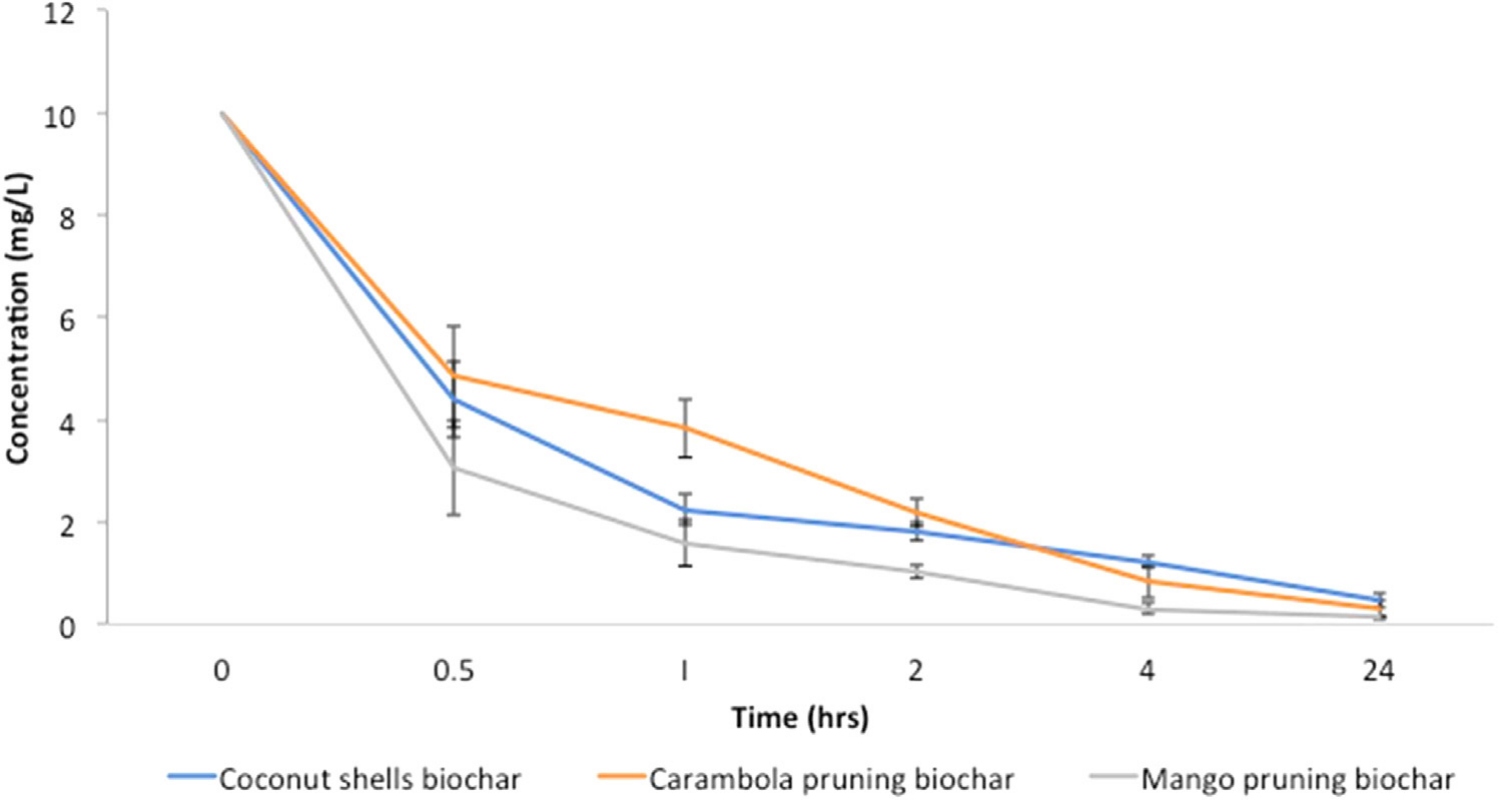
Adsorption capacity of fentin acetate using different types of biochar.

**Fig. 7:**
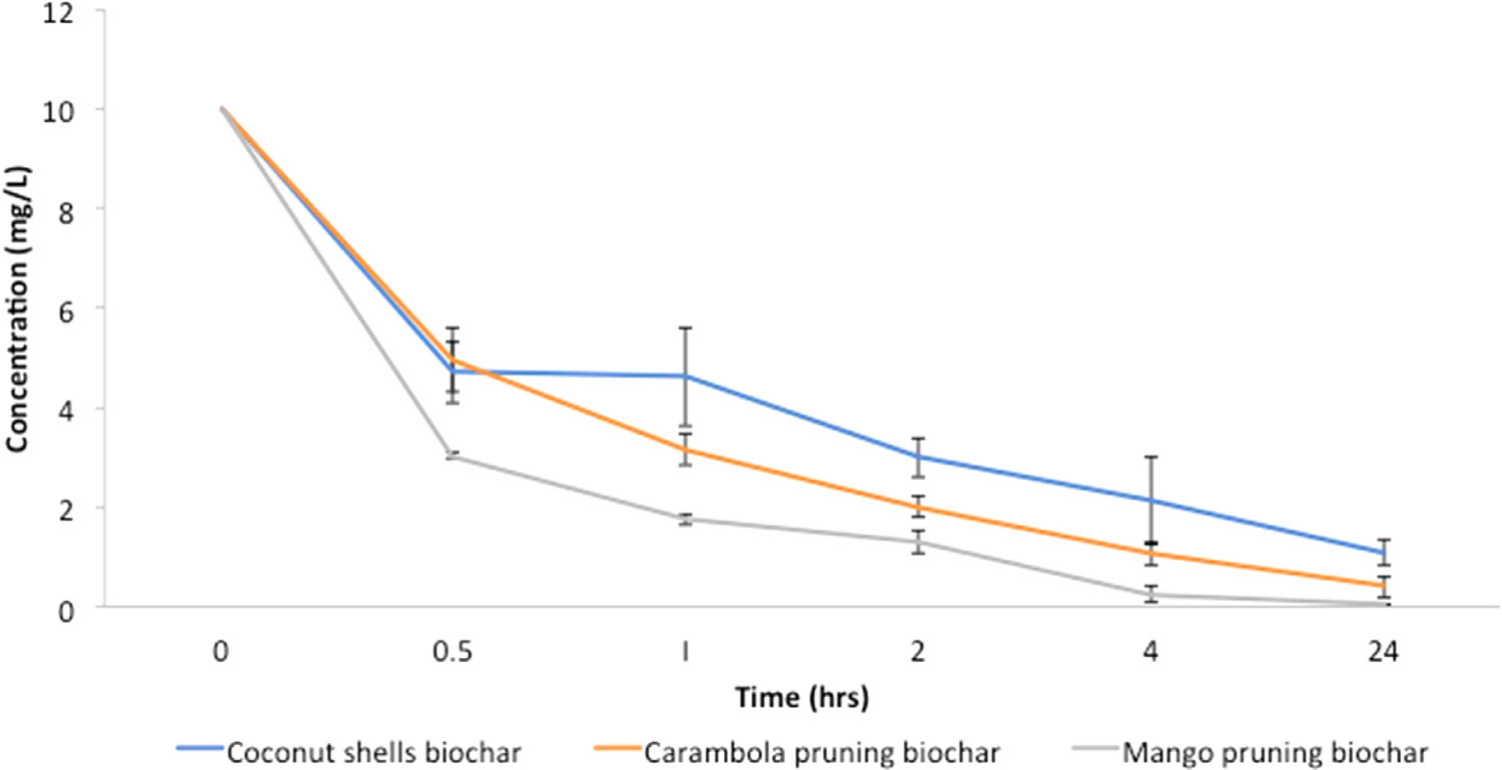
Adsorption capacity of Cypermethrin using different types of biochar.

The data included in this study incorporate a design of a small-scale pyrolyser to accommodate the management of farm waste and the characterisation of the biochar produced. The study was conducted at the Malaysian Agricultural Research and Development Institute (MARDI) headquarters in Serdang, Selangor, Malaysia. The data have been organised into seven figures and three tables. The two main products used in the pyrolysis were biochar and pyroligneous liquid (wood vinegar). The raw materials used were coconut shells, carambola pruning, and mango pruning. Fig. 1, Fig. 2, Fig. 3, Fig. 4 show the design of the pyrolyser used in this study, with four (4) perspectives showing the top view, front view, side view, and rear view, as well as the design measurements and the actual view of the pyrolyser after completion. The data can be accessed from the data files entitled “All views.png”, “Front views.png”, “Design measurements.png”, and “Actual view.png” in the Mendeley repository. Meanwhile, Table 1, Table 2, Table 3 show the elemental composition, physicochemcial properties, calorific values, and porosity analysis of the biochar from the three biomasses used in the study: (a) coconut shells, (b) carambola pruning, and (c) mango pruning. The data for these analyses can be accessed from the data files entitled “CHNS Analyses.xlsx”, “Physicochemical and calorific value.xlsx”, and “Porosity BET.xslx” in the Mendeley repository. The data indicated that the elemental composition of the raw materials was comparable with that of other materials [1,2]. Meanwhile, the carbon content of the biochar was higher than that of the raw materials and surpassed 50% elemental composition, indicating that the product materials (biochar) conserved high percentages of carbon. Table 3 shows that the biochar from the carambola pruning had the highest surface area characteristics. Fig. 5 shows the SEM analysis of the biochar produced from these three sources. This figure also highlights uniform and visibly structured hollow pores within the biochar obtained from the carambola pruning. For further reference, the SEM figures can be accessed in the file “SEM Biochar.png”, which is in the Mendeley repository. Further characterisation studies were undertaken. Fig. 6, Fig. 7 show the results from the study of the adsorption characteristics of the biochar, which were obtained using two different agricultural pesticides, a) fentin acetate and b) cypermethrin. The purpose of the study was to evaluate how effectively the biochar would remove the recalcitrant pesticide from the effects of agricultural run-off [3]. The results show that all three sources of biochar demonstrated effective adsorption capacity in a 24-hour adsorption study, based on an initial pesticide concentration of 10 mg/L. A significant adsorption result was obtained for the biochar made from mango pruning. The data for these analyses were kept in the file entitled “Adsorption study.xlsx” in the Mendeley repository.

**Table 1:** Elemental composition of raw materials and biochar produced from different agricultural wastes.

Materials	N [%]	C [%]	H [%]	S [%]	C/N ratio
Carambola pruning (raw)	0.84 ± 0.06	45.46 ± 0.50	6.42 ± 0.35	0.22 ± 0.01	54.21 ± 4.43
Mango pruning (raw)	0.64 ± 0.02	47.44 ± 0.92	6.21 ± 0.48	0.22 ± 0.03	73.58 ± 0.73
Coconut shells (raw)	0.27 ± 0.04	50.85 ± 0.23	6.18 ± 0.17	0.24 ± 0.01	188.27 ± 25.13
Carambola pruning (biochar)	1.033 ± 0.08	71.30 ± 2.03	1.82 ± 0.21	1.29 ± 0.98	69.18 ± 3.19
Mango pruning (biochar)	0.66 ± 0.19	76.66 ± 3.02	2.38 ± 0.20	0.14 ± 0.04	123.88 ± 38.53
Coconut shells (biochar)	0.25 ± 0.03	71.10 ± 1.19	6.15 ± 1.81	0.22 ± 0.06	284.89 ± 39.80

**Table 2:** Physicochemical characteristics and calorific values of biochar produced from different agricultural wastes.

Biochar samples	Carambola pruning (biochar)	Mango pruning (biochar)	Coconut shells (biochar)
pH	9.96 ± 0.01	9.54 ± 0.01	8.98 ± 0.06
Electrical conductivity (uS)	3460.00 ± 72.11	2230.00 ± 278.75	1098.33 ± 41.10
Ash (%)	13.51 ± 0.09	8.81 ± 0.61	2.38 ± 0.19
Calorific value (Cal/g)	5709.27 ± 11.52	6069.60 ± 169.43	6926.37 ± 46.24

**Table 3:** Porosity properties of biochar.

Biochar samples	Carambola pruning	Mango pruning	Coconut shells
S_BET_ (m^2^/g)	57.15	12.65	4.44
V_total_ (cm^3^/g)	0.0148495	0.0002775	0.000078
Average pore size (nm)	0.9990	0.2626	0.0814

## 4 Experimental Design, Materials and Methods

### 4.1 Pyrolyser design and development

The pyrolysis system used in this study comprised a two-stage production chamber with 10 kg of capacity for the initial raw materials (Fig. 1, Fig. 2, Fig. 3, Fig. 4). The main chamber produced biochar, while the second chamber, also known as the condensation chamber, produced pyroligneous liquid or wood vinegar through the condensation of white smoke, a by-product of the pyrolysis process. The biochar production chamber design was based on a previous study involving a bottom-up self-heating pyrolysis method practised by farmers [4,5]. Meanwhile, the condensation chamber design was based on the cyclonic concept and aimed to optimise the amount of pyroligneous liquid collected. According to [6], the cyclone technique used to be the predominant way to remove particles from gas streams. Data about the utilisation of pyroligneous liquid or wood vinegar was reported in [7]. The pyrolyser was designed and drawn using SolidWorks software version 2020. Having finalised the design, fabrication was outsourced to a third party. The main production chamber was built using stainless steel, while the condensation chamber was built using a mixed-steel material. The design and drawing were introduced in [8] as a means of disseminating information among local farmers and stakeholders. It was registered as copyrighted work for public goods (ref no: CR2022/40/105) and made free for public access and reuse.

### 4.2 Collection of raw materials and pyrolysis set-up

The three biomasses used in this study - coconut shells, carambola pruning, and mango pruning - were obtained from local sources. The coconut shells were obtained from a local wet market in Serdang, Selangor, Malaysia, while the carambola and mango pruning were obtained from research plots at MARDI headquarters, Serdang, Selangor, Malaysia. The pyrolysis was undertaken using a self-heating process, whereby a small amount of biomass (e.g., coconut shells) was ignited before a chain of charring process occurred. The system underwent pyrolysation without depending on external heat sources [4]. For a batch of 10 kg capacity input in the biochar chamber, pyrolysis occurred slowly for between one and two hours. Carbonisation occurred under an uncontrolled temperature, whereby the carbonised biomass itself would provide the heat required for a pyrolysis chain to continue. To measure the temperature inside the pyrolysis chamber, a portable thermocouple was locally fabricated and assembled (TH Muda Engineering) before being used. The recorded temperatures ranged from 195 °C at the beginning to reach a maximum of 667 °C. These data were previously reported in [5]. Since the previous development, the design of the condensation chamber has been improved to enhance the collection of pyroligneous liquid or wood vinegar (Fig. 1, Fig. 2, Fig. 3). Additionally, low-temperature pyrolysis was defined as temperatures within this range of pyrolysis temperatures [9], [10], [11]. The upper part of the condensation chamber had a small outlet whose function was to release excess white smoke (Fig. 4). In conventional farmers’ practice (although this has yet to be documented), the white smoke has the beneficial function of repelling pests on their farms. By observation, the visible smoke normally disappeared within a three-metre radius of the chamber.

### 4.3 Data collection and analysis for elemental composition, physicochemical, calorific value, and surface area characterisation

The powdered raw materials and biochar samples (triplicate) were taken for nutrient analysis to determine the percentages of the carbon, hydrogen, nitrogen, and sulphur elements. For each sample, 30 mg of the substrate was mixed with 30 mg of tungsten and wrapped in metal foil. The samples were then analysed using an elemental analyser (vario MACRO cube). Meanwhile, the pH and electrical conductivity (EC) were analysed using the Eutech P700 (Eutech Instruments), while the ash content (percentage) was determined using a standard ashing method through incineration in a muffle furnace at 500 °C. Calorific content data were obtained by outsourcing the analysis to an accredited laboratory, MARDI Laboratory (MARDILab) at Serdang, Selangor, Malaysia. Additionally, the BET surface characteristics were determined using Micromeritics® Tristar II Plus. This analysis was carried out at the Universiti Putra Malaysia (UPM) laboratory. The chemical structure was analysed using SEM (FEI Quanta 400). The SEM analysis was carried out at the Universiti Kebangsaan Malaysia (UKM) laboratory. The SEM data presented in this study are shown at one hundred (100X) and five hundred times magnification (500X).

### 4.4 Adsorption characteristics of biochar using fentin acetate and cypermethrin

The adsorption study was conducted using a standard jar test method, whereby initial concentrations of 10 mg/l of each pesticide compound solution (from fentin acetate and cypermethrin) were prepared separately. Into the jars containing a pesticide concentration solution were added 0.1 g of biochar sample (in triplicate) from either the coconut shells, carambola pruning, or mango pruning. Following this, the mixed samples and pesticide solutions were shaken vigorously in an incubator shaker. The mixed solutions were sampled at the intervals of 0, 0.5, 1, 2, 4, and 24 h. For each sampling process, the concentrations of fentin acetate and cypermethrin were determined using liquid chromatography–mass spectrometry (LC-MS/MS) (Sciex 5500).

### 4.5 Data analysis

The elemental composition, physicochemical, and calorific values data were analysed using descriptive statistical analyses, whereby the mean and standard deviation (SD) values were calculated using Microsoft Office Excel (MS Excel). Meanwhile, the statistical model for the adsorption study was determined using the statistical analysis system (SAS) version 9.3 (SAS Institute, Inc., USA). An analysis of variance (ANOVA) was conducted, whereby different sources of biochar (treatment) were compared using a Duncan's test.

## Limitations

This design could only accommodate small-scale biochar production at 10 kg per batch of initial raw materials. Therefore, in order to evaluate the quality of the biochar produced for validation purposes, additional analysis is required of the data characteristics obtained from a larger design that can process over 10 kg of initial raw materials. In addition, the current study focused on only one location as a source of raw materials. In the future, further studies should be conducted using raw materials from different location sources so that more datasets can be obtained. Overall, the data from this study can be used as a benchmark for future characterisation studies.

## Ethics Statement

This work involved no human or animal studies, so an ethics statement is not applicable.

## CRediT authorship contribution statement

**Mohammad Shahid Shahrun:** Conceptualization, Methodology, Formal analysis. **Mohammad Hariz Abdul Rahman:** Project administration, Methodology, Formal analysis, Writing – original draft, Writing – review & editing. **Nur Adliza Baharom:** Methodology, Formal analysis. **Fauzi Jumat:** Methodology, Formal analysis. **Mohamad Jani Saad:** Visualization, Investigation. **Mohd Fazly Mail:** Methodology, Formal analysis. **Norziana Zin Zawawi:** Methodology, Funding acquisition. **Farah Huda Sjafni Suherman:** Project administration, Funding acquisition.

## Declaration of Competing Interest

The authors declare that they have no known competing financial interests or personal relationships that could have appeared to influence the work reported in this paper

## Data Availability

Design of a pyrolysis system and the characteristics of biochar produced from selected agricultural wastes (Original data) (Mendeley Data)
